# A Healthy Heart and a Healthy Brain: Looking at Mitophagy

**DOI:** 10.3389/fcell.2020.00294

**Published:** 2020-05-06

**Authors:** Hongke Luo, Ruohan Zhang, Judith Krigman, Allison McAdams, Serra Ozgen, Nuo Sun

**Affiliations:** ^1^Department of Physiology and Cell Biology, College of Medicine, The Ohio State University Wexner Medical Center, Columbus, OH, United States; ^2^Dorothy M. Davis Heart and Lung Research Institute, The Ohio State University Wexner Medical Center, Columbus, OH, United States; ^3^Department of Graduate Research, College of Pharmacy, The Ohio State University, Columbus, OH, United States

**Keywords:** mitophagy, neurodegenerative diseases, cardiovascular disorders, mitochondrial, autophagy

## Abstract

Mitochondrial dysfunction is a hallmark of aging and is a major contributor to neurodegenerative diseases and various cardiovascular disorders. Mitophagy, a specialized autophagic pathway to remove damaged mitochondria, provides a critical mechanism to maintain mitochondrial quality. This function has been implicated in a tissue’s ability to appropriately respond to metabolic and to bioenergetic stress, as well as to recover from mitochondrial damage. A global decline in mitophagic flux has been postulated to be linked to pathological alterations that occur in the heart and the brain as well as a general age-dependent decline in organ function. Cellular observation suggests multiple mechanistically distinct pathways converge upon and activate mitophagy. Over the past decade, additional molecular components within mitophagy have been discovered, including several disease-associated genes that are functionally implicated in mitophagy. However, the pathophysiological role of mitophagy, and how it is regulated within normal physiology or various disease states, is less well established. Here, we will review the evidence that a decline in mitophagy contributes to impaired mitochondrial homeostasis and may be particularly detrimental to postmitotic neurons and cardiomyocytes. We will discuss mitophagy’s pathological significance in both neurodegenerative diseases and cardiovascular disorders. Additionally, signaling pathways regulating mitophagy are reviewed, with emphasis placed on how these pathways might contribute to disease progression. Understanding mitophagy’s role in the mechanisms of disease pathogenesis should allow for the development of more efficient strategies to battle pathological conditions associated with mitochondrial dysfunction.

## Introduction

Autophagy is the regulated process that targets unnecessary or dysfunctional cellular components for lysosomal-mediated removal (Mizushima et al., 1998; Klionsky and Emr, 2000; Kim and Lee, 2014; Yan and Finkel, 2017). This process is a crucial recycling mechanism to maintain cellular homeostasis under normal physiological states as well as in disease conditions as it facilitates the orderly degradation of damaged organelles and other cellular components, (Levine, 2005; He et al., 2012; Kim and Lee, 2014; Fernández et al., 2018). The removal of mitochondria through autophagy, a process called mitophagy, is an important element of mitochondrial quality control (Youle and Narendra, 2011; Palikaras et al., 2018). This system mediates the selective engulfment of defective or damaged mitochondria by autophagosomes and their subsequent catabolism by lysosomes, preserving overall mitochondrial homeostasis (Youle and Narendra, 2011; Palikaras et al., 2018). Significant evidence suggests that mitophagy may be required for adaptation to various stresses, and that dysregulation of mitophagy may contribute to a host of diseases, most notably neurodegenerative conditions such as Parkinson’s disease (PD) (Narendra et al., 2008; Geisler et al., 2010; Lazarou et al., 2015). The products of two PD-associated genes, the phosphatase and tensin homolog (PTEN)-induced putative kinase 1 (PINK1) and the cytosolic E3 ligase Parkin, can sense mitochondrial damage and mediate ubiquitin-dependent mitophagy (Narendra et al., 2008; Youle and Narendra, 2011; Pickrell and Youle, 2015; Yamano et al., 2016; Palikaras et al., 2018). Loss-of-function mutations in the regulatory kinase PINK1 and the ubiquitin ligase Parkin have each been identified as a cause for familial, early-onset PD, suggesting that impaired mitophagy may contribute to the loss of dopaminergic neurons that occurs during the PD disease progression (Kitada et al., 1998; Valente et al., 2004; Lazarou et al., 2015; Palikaras et al., 2017; Harper et al., 2018). Similarly, the preservation of mitochondrial function through mitophagy is also important for mitochondria-rich and bioenergetically demanding organs such as the heart (Murphy et al., 2016; Bravo-San Pedro et al., 2017). Cardiac mitophagy may be required for the myocardium to recover from mitochondrial damage that occurs, for instance, during cardiac hypertrophy or ischemic injury (Murphy et al., 2016; Bravo-San Pedro et al., 2017). Evidence suggests a decline in mitophagy may contribute to the myriad of pathological events that occur under metabolic stress or in the elderly heart (Eisenberg et al., 2016; Leon and Gustafsson, 2016; Lesnefsky et al., 2016; Murphy et al., 2016; Shirakabe et al., 2016a, b).

**FIGURE 1:**
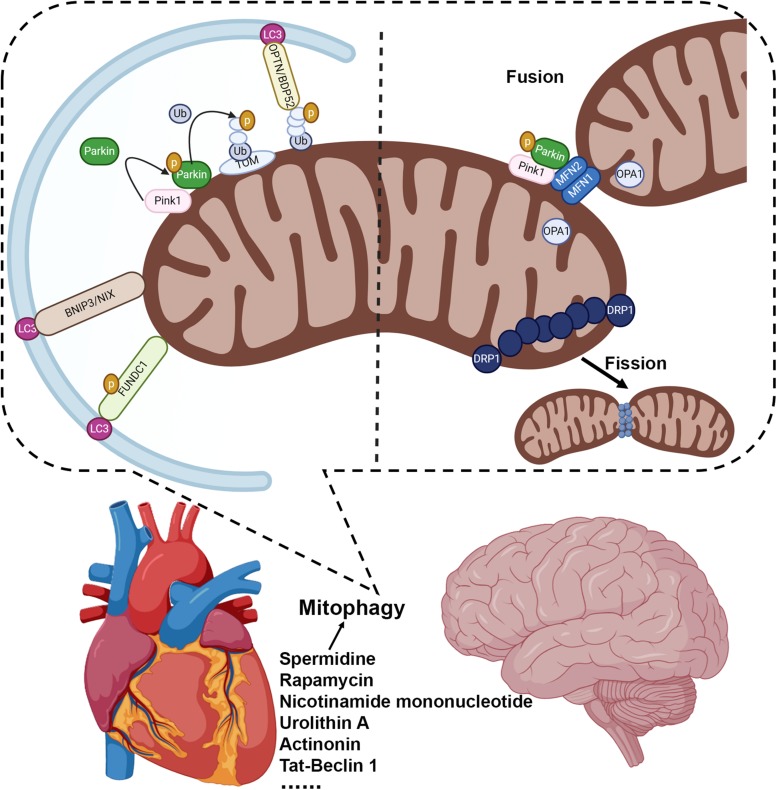
Mitophagy is a critical mechanism to maintain mitochondrial quality in the brain and heart. Mitophagy can be regulated by multiple mechanistically distinct pathways: the PINK1/Parkin dependent mitophagy, which is mediated by mitophagy receptors such as optineurin and NDP52; BNIP3 and NIX regulated mitophagy to facilitate autophagic engulfment of mitochondria; and FUNDC1 mediated mitophagy. Manipulating autophagy/mitophagy activity may preserve mitochondrial function and serve as a novel therapeutic strategy, such as spermidine (Eisenberg et al., 2016), rapamycin (Sciarretta et al., 2012; Yan et al., 2013; Dai et al., 2014; Ranek et al., 2019), nicotinamide mononucleotide (Fang et al., 2019), urolithin A (Ryu et al., 2016; Fang et al., 2019), actinonin (Fang et al., 2019), and Tat-Beclin 1 (Shoji-Kawata et al., 2013; Shirakabe et al., 2016b).

Considerable interest has been focused on elucidating the molecular mechanisms of mitophagy, particularly on PINK1/Parkin’s ability to catalyze the reaction to ubiquitinate a range of outer mitochondrial membrane (OMM) proteins (Narendra et al., 2008, 2010; Matsuda et al., 2010; Lazarou et al., 2015; Palikaras et al., 2017; Harper et al., 2018). PINK1 is a mitochondrial-targeted kinase whose stability is regulated, at least in part, by mitochondrial membrane potential (MMP) (Narendra et al., 2008, 2010; Matsuda et al., 2010; Lazarou et al., 2015). When MMP is dissipated following mitochondrial damage, PINK1 accumulates on the OMM, where it can phosphorylate ubiquitin attached to OMM and recruit Parkin to impaired mitochondria, thereby triggering its latent E3 ubiquitin ligase activity to ubiquitinate OMM proteins (Narendra et al., 2008, 2010; Matsuda et al., 2010; Lazarou et al., 2015). This post-translational tagging of OMM proteins with ubiquitin can serve as a signal to recruit receptors such as optineurin and NDP52, which act as a signal facilitating autophagosomal engulfment of individual damaged mitochondria (Itakura et al., 2012; Lazarou et al., 2015) (Figure 1). PINK1/Parkin-independent mechanisms can also initiate mitophagy (Novak et al., 2010; Hanna et al., 2012). The proapoptotic Bcl2 family proteins Nix and Bnip3 on the OMM participate in autophagic engulfment of mitochondria through direct interaction with LC3 on autophagosomes (Novak et al., 2010; Hanna et al., 2012) (Figure 1). Newly formed erythrocytes, also known as reticulocytes, eliminate their membrane-bound organelles, including mitochondria, during the course of development (Koury et al., 2005; Schweers et al., 2007). Nix regulated mitophagy may contribute to the rapid and coordinated clearance of mitochondria, also called the programmed mitochondrial degradation, in the process of erythropoiesis, whereas loss of Nix in mice impairs mitochondrial degradation during erythroid maturation (Schweers et al., 2007; Sandoval et al., 2008). The OMM protein FUNDC1 (FUN14 domain containing 1) can also bind LC3 to recruit autophagosome and promote mitophagy (Liu et al., 2012; Chen et al., 2016) (Figure 1). FUNDC1-mediated mitophagy may depend on its phosphorylation status regulated by the Unc-51 Like Autophagy Activating Kinase 1 (ULK1), casein kinase 2 (CK2), and PGAM5 phosphatase (Chen et al., 2014, 2016; Palikaras et al., 2018). Recent progress has demonstrated a crucial role for the inner mitochondrial membrane (IMM) proteins in mitophagy regulation. Notably, IMM protein prohibitin 2 (PHB2), a component of the mitochondrial prohibitin complex, may serve as a mitochondrial receptor for mitophagy upon mitochondrial depolarization (Wei et al., 2017).

While there has been considerable mechanistic insight into the regulatory pathways of mitophagy, investigation into the role of mitophagy in healthy and disease conditions has only just begun. Mouse tissue analysis has revealed a variation in basal mitophagy levels (Sun et al., 2015; McWilliams et al., 2018b), which might be required for continuous mitochondrial housekeeping. Stressed-induced mitophagy has been implicated in a tissue’s ability to recover from mitochondrial damage, as well as appropriately responding to metabolic and bioenergetic stressors (Sun et al., 2016; Bravo-San Pedro et al., 2017; Palikaras et al., 2018). In energetically demanding tissues such as the heart and the brain, the mitophagic removal might require exquisite regulatory mechanisms, which merit further investigation. Additionally, recent studies have demonstrated an essential role for programmed mitophagy that directs the developmental metabolic transitioning of cardiac mitochondria (Gong et al., 2015). In summation, the preservation of mitochondrial function is crucial for all tissues, but it is undoubtedly critical for bioenergetically demanding organs such as the brain and the heart. Here, we will review how mitophagy represents a major pathway to help sustain mitochondrial quality and how alterations in mitophagy can contribute significantly to both neurodegenerative and cardiovascular diseases (Figure 1). The ability to genetically and pharmacologically modulate mitophagic flux may provide considerable insight into mitochondria related disease mechanisms and allow for the development of novel therapeutic approaches.

## Mitophagy in Neurodegenerative Diseases

There is a growing appreciation regarding the critical role of altered mitochondrial function in the pathogenesis of neurodegenerative diseases (Valente et al., 2004; Youle and Narendra, 2011; Subramaniam and Chesselet, 2013; Hsieh et al., 2016; Palikaras et al., 2018). Selective identification and removal of damaged or dysfunctional mitochondria through mitophagy is thought to be an effective mechanism in maintaining neuronal mitochondrial homeostasis (Hwang et al., 2015; Hsieh et al., 2016; McWilliams et al., 2018b). Evidence suggests a decline in mitophagy might contribute to many neurodegenerative diseases (Burchell et al., 2013; Bingol et al., 2014; Fivenson et al., 2017; Fang et al., 2019).

Attention to mitophagy has been augmented by its link to genes related to inherited forms of early-onset PD (Kitada et al., 1998; Valente et al., 2004; Lazarou et al., 2015; Bingol and Sheng, 2016; Sliter et al., 2018). PD is one of the most common neurodegenerative disorders characterized by a series of motor impairments including tremors, rigidity, bradykinesia (slowness of movement), and postural instability (poor balance), which are caused by the progressive loss of dopaminergic neurons in the substantia nigra of the brain (Ascherio and Schwarzschild, 2016). Although medical and surgical treatments may provide symptomatic relief, there is no cure for PD (Deep-Brain Stimulation for Parkinson’s Disease Study Group, 2001; Narendra et al., 2009; Gao et al., 2011; Brod et al., 2012; Richard et al., 2012; Burchell et al., 2013; Hauser et al., 2013; Olanow et al., 2014; Gan-Or et al., 2015; Ascherio and Schwarzschild, 2016). Identification of genes mutated in monogenic forms of PD has provided valuable insight into the etiology of the disease (Narendra et al., 2009; Burchell et al., 2013; Gan-Or et al., 2015; Ascherio and Schwarzschild, 2016). Specifically, mutations or variants of PINK1 and Parkin have been found in the inherited forms of early-onset PD’s patient (Kitada et al., 1998; Valente et al., 2004; Lazarou et al., 2015; Bingol and Sheng, 2016; Sliter et al., 2018). Biochemical and genetic studies reveal the products of these two genes, PINK1 and Parkin, normally function within the same genetic pathway to govern mitochondrial quality control (Lazarou et al., 2015; Bingol and Sheng, 2016; Sliter et al., 2018). PINK1 can detect and accumulate on the damaged mitochondria, which results in activation of Parkin’s E3 ubiquitin ligase activity and recruitment of Parkin to the dysfunctional mitochondrion (Narendra et al., 2008; Matsuda et al., 2010; Hasson et al., 2013; Lazarou et al., 2015). Parkin then ubiquitinates OMM proteins to promote mitophagy (Hasson et al., 2013; Lazarou et al., 2015; Sliter et al., 2018). The realization that PINK1 and parkin can work together in the same pathway to coordinate mitophagy strengthens the notion that mitophagy may play an important role in PD (Hasson et al., 2013; Lazarou et al., 2015; Sliter et al., 2018). Therefore, the biochemical mechanisms of PINK1/Parkin mediated mitophagy, along with their roles in various models of PD, have been studied extensively over the past 10 years. While the generation of Parkin and PINK1 mutant flies has elucidated the functions of these genes in regulating mitochondrial integrity (Clark et al., 2006; Park et al., 2006; Palikaras et al., 2018), PINK1 and Parkin knockout mouse models poorly recapitulate dopamine neurodegeneration and the pathophysiology of human PD (Goldberg et al., 2003; Itier et al., 2003; Kitada et al., 2007; Billia et al., 2011; Hasson et al., 2013; Lazarou et al., 2015; Sliter et al., 2018). A significant portion of basal mitophagy occurs within the PD-relevant dopamine neurons of the substantia nigra pars compacta and in microglia, indicating a critical role of mitophagy in these cells under physiological conditions (McWilliams et al., 2018b). However, PINK1 or Parkin does not appear to influence basal mitophagy (McWilliams et al., 2018a, b). This data suggests that the precise role of PINK1/Parkin under physiological conditions remains to be defined. The pathophysiological basis of the cross-regulation between various mitochondrial quality control pathways will be of prime importance in understanding how mitophagy occurs and how it relates to PD progression.

Insight into the mechanisms underlying mitophagy and the importance of mitochondrial quality control have extended to other common neurodegenerative diseases associated with mitochondrial dysfunction such as Alzheimer’s disease (AD), Huntington’s disease (HD), and amyotrophic lateral sclerosis (ALS) (Trushina et al., 2004; Ye et al., 2015; Palikaras et al., 2017; Chakravorty et al., 2019; Evans and Holzbaur, 2019). Impaired mitophagy is closely related to AD, another important age-related neurodegenerative disease (Fivenson et al., 2017; Fang et al., 2019). AD is an irreversible, progressive brain disorder, characterized by cognitive defects and a progressive decline in memory (Fivenson et al., 2017; Fang et al., 2019). Like PD, the symptoms of AD are related to the loss of important neurons in certain areas of the brain, including the hippocampus, entorhinal cortex, temporal lobe, parietal lobe, and frontal lobe (Fivenson et al., 2017; Fang et al., 2019). Although the etiology of AD remains unclear, the accumulation of intracellular hyperphosphorylated tau (p-tau) and extracellular amyloid β-peptide (Aβ) have been identified in the onset and progression of the disease (Braak and Braak, 1995; Fivenson et al., 2017; Fang et al., 2019; Thal et al., 2019). Aβ and p-tau pathologies can contribute to mitochondrial defects, and in AD, neurons may display mitochondrial dysfunction and a bioenergetic deficit (Mattson et al., 2008; Kapogiannis and Mattson, 2011; Fang et al., 2019). Impairment of mitochondrial function has been identified in the brain tissues of AD mouse models, as well as in human samples of both sporadic and familial types of AD (Lustbader et al., 2004; Fang et al., 2019). Mitochondria dysfunction can accelerate Aβ deposit at the cellular level and contribute to hyperphosphorylation of tau in neurons (Esposito et al., 2006; Mattson et al., 2008; Kapogiannis and Mattson, 2011; Fang et al., 2019). Accumulating evidence demonstrates that a decline in mitophagy may contribute to impaired mitochondrial homeostasis in AD (Fivenson et al., 2017; Chakravorty et al., 2019; Fang et al., 2019). Under AD-linked pathophysiological conditions, Parkin-mediated mitophagy is altered in AD mutant neurons and in AD patient brains (Ye et al., 2015). Inadequate mitophagy capacity may contribute to the aberrant accumulation of dysfunctional mitochondria in AD-affected neurons (Ye et al., 2015; Fivenson et al., 2017; Chakravorty et al., 2019; Fang et al., 2019). Interestingly, using postmortem human AD brain samples, induced pluripotent stem cell-derived human AD neurons, and a set of AD animal models, a recent study demonstrates defective mitophagy in AD (Fang et al., 2019). Of note, pharmacological restoration of mitophagy ameliorates memory loss in both *Caenorhabditis elegans* and mouse models of AD (Fang et al., 2019). Thus, targeting the mitochondrial quality control system may provide a novel therapeutic strategy for AD.

Mitochondrial dysfunction also has been implicated in the pathology of HD, another progressive neurodegenerative disease caused by a genetic mutation in the huntingtin gene (Shirendeb et al., 2011; Hwang et al., 2015). In HD, the pathological expansion of CAG trinucleotide repeat encoding a polyglutamine (polyQ) tract in the amino-terminal region of the Huntingtin protein results in an abnormal polyglutamine stretch and in accumulation of polyQ-expanded HTT (Mattson et al., 2008; Song et al., 2011; Martin et al., 2015). Due to the loss of GABAergic neurons in the basal ganglia HD is characterized by motor dysfunction, psychiatric disturbance, and a decline in cognition (Ross and Tabrizi, 2011). The mutant huntingtin protein may affect a wide range of cellular pathways and functions, including transcriptional regulation, axonal trafficking of vesicles, mitochondrial function, ubiquitin-mediated proteolysis, and the autophagic systems (Luthi-Carter and Cha, 2003; Gauthier et al., 2004; Trushina et al., 2004; Wong and Holzbaur, 2014; Martin et al., 2015). Damaged mitochondria and impaired mitophagy are closely related to neuronal death and disease progression in HD (Mattson et al., 2008; Song et al., 2011; Martin et al., 2015). Mutant HTT can bind the mitochondrial fission GTPase dynamin−related protein−1, and perturb mitochondrial dynamics in HD, leading to the accumulation of damaged mitochondria and increased reactive oxygen species (ROS) (Shirendeb et al., 2011; Song et al., 2011). Research has indicated that mutant HTT mediated transcriptional dysregulation of autophagy-related genes, cargo recognition failure of autophagosomes, and impaired trafficking of lysosomes contribute to inefficient autophagy in HD (Martinez-Vicente et al., 2010). Additionally, recent studies demonstrate that mutant HTT can interact with the Ser/Thr-kinase ULK1 and p62/SQTM12 (Lim et al., 2015; Wold et al., 2016), thereby potentially regulating mitophagy. Furthermore, expression of mutant HTT with expanded polyglutamine repeats may alter GAPDH-mediated mitophagy, thus contributing to the pathology of HD (Hwang et al., 2015). Using mt-Keima mice to measure *in vivo* mitophagic flux, markedly reduced levels of mitophagy have been observed in mutant HTT-expressing mice (Sun et al., 2015). Therefore, the presence of the expanded polyQ tract can affect the neuronal mitophagy, and consequently promote mitochondrial dysfunction, contributing to disease progression in HD.

Defects in mitophagy appear to have significance with regards to familial ALS, also known as motor neuron disease or Lou Gehrig’s disease (Evans and Holzbaur, 2019). ALS is characterized by a progressive degeneration of motor neurons in the spinal cord and brain (Evans and Holzbaur, 2019). Several of the ALS-associated genes have been functionally implicated in autophagy or the mitophagy process (Evans and Holzbaur, 2019). These include VCP, encoding valosin-containing protein, implicated in autophagy (Buchan et al., 2013) and the maintenance of lysosomal function (Papadopoulos et al., 2017), as well as the noncoding sequence of the C9ORF72 gene, mutations of which account for approximately 40% of familial ALS (DeJesus-Hernandez et al., 2011; Renton et al., 2011; Floeter et al., 2017). C9ORF72 may regulate ULK1 and may function in the regulation of lysosomal fusion or function (DeJesus-Hernandez et al., 2011; Renton et al., 2011; Floeter et al., 2017). Mutations in the serine/threonine kinase Tank-binding kinase 1 (TBK1) and the autophagy receptor optineurin (OPTN) are associated with ALS (Moore and Holzbaur, 2016). ALS-linked mutations in OPTN and TBK1 can interfere with mitophagy suggesting, an inefficient turnover of damaged mitochondria may represent a key pathophysiological mechanism contributing to neurodegenerative disease. The TBK1-OPTN axis can target damaged mitochondria and promote autophagosome formation around mitochondria triggering mitophagy (Lazarou et al., 2015; Palikaras et al., 2017; Harper et al., 2018). Inhibition of TBK1 or expression of ALS-linked TBK1 mutant can block efficient autophagosome formation (Moore and Holzbaur, 2016). In neurons, an ALS-associated mutation in OPTN is sufficient to disrupt mitochondrial function under basal conditions, due to the slow kinetics of mitophagy (Evans and Holzbaur, 2020). Mutant Cu/Zn superoxide dismutase (SOD1) also causes alterations of mitochondrial function in ALS (Palomo et al., 2018). Interestingly, a recent study demonstrates that Parkin genetic ablation slows down motor neuron demise, delays ALS disease progression, and thus extends the lifespan of the SOD1−G93A mutant mice (Palomo et al., 2018). Therefore, understanding the molecular basis for autophagy and mitophagy in familial ALS should provide considerable insight into the disease-causing mechanisms involved in ALS pathogenesis, and may lead to the development of more effective therapeutic approaches for ALS.

Mitochondrial defects have been linked to neuronal dysfunction and the pathogenesis of neurodegenerative diseases. In neurons, mitophagy serves as a major pathway required for the preservation of mitochondrial function. Mitophagy deficit’s role in neurodegenerative diseases has only been recently recognized despite the significant advancements in understanding the molecular and the cellular mechanisms that govern mitophagy. Given the fact that neurons have an exceptionally high demand for ATP, the quality control of mitochondria is essential for neuronal functions. The same can be said for cardiomyocytes, in which mitochondria occupy approximately one-third of the cell volume (Schaper et al., 1985; Murphy et al., 2016; Bertero and Maack, 2018). Therefore, defective mitophagy may impair mitochondrial homeostasis and can be particularly detrimental for these terminally differentiated cells such as the postmitotic neurons and cardiomyocytes (Youle and Narendra, 2011; Bravo-San Pedro et al., 2017; Palikaras et al., 2018). Interestingly, researchers recently have noticed a link between various cardiovascular abnormalities and neurodegenerative diseases. For instance, Aβ aggregations have been reported to be present in the hearts of patients with idiopathic dilated cardiomyopathy (Gianni et al., 2010). And recent studies have demonstrated compromised myocardial function and intramyocardial deposits of Aβ in AD patients (Troncone et al., 2016). Likewise, clinical studies on HD patients at various stages of disease progression have revealed a high incidence of cardiovascular events (Melik et al., 2012; Stephen et al., 2015; Kobal et al., 2017). Although neurons and cardiomyocytes vary a lot in their structure and function, these findings depict a possible biological framework linking neurodegenerative diseases to cardiovascular risks. However, evidence of a possible role that mitochondria play calls for further analysis of this connection.

## Mitophagy in Cardiovascular Disease

The heart demands a substantial amount of energy to fuel myocardial contraction and is subsequently rich in mitochondria (Murphy et al., 2016; Bertero and Maack, 2018). The cardiac mitochondria account for ∼30% of myocellular volume and possess the capacity to use multiple metabolic substrates to generate ATP under a wide range of physiological and pathological conditions (Schaper et al., 1985; Murphy et al., 2016; Bertero and Maack, 2018). Also, mitochondria are essential organelles in the regulation of metabolic substrate utilization, cell death, calcium storage, and ROS levels (Wallace et al., 2010; Mishra and Chan, 2016; Sun et al., 2016). Mitophagy plays a critical role in preserving mitochondrial quality in normal cardiovascular physiology and in pathological circumstances. During the early perinatal period, changes in oxygen and nutrient availability catalyze a switch in cardiac substrate preference from glucose to fatty acids (Gong et al., 2015; Dorn, 2016). Recent studies have demonstrated an essential role for mitophagy in the normal perinatal transformation of myocardial metabolism (Gong et al., 2015). During cardiac stress and injury, mitophagy increases to help clear damaged mitochondria, as well as prevent oxidative damage and cell death (Hoshino et al., 2013; Kubli et al., 2013). In response to pressure overload-induced mitochondrial dysfunction, mitophagy can be activated dynamically and play a protective role against heart failure (Shirakabe et al., 2016b). Over the past decade, significant progress has been made in understanding of the physiological and pathological roles of cardiac mitophagy (Billia et al., 2011; Murphy et al., 2016; Tong and Sadoshima, 2016; Bravo-San Pedro et al., 2017; Wang et al., 2018).

The functional significance of cardiac mitophagy has been revealed by analyzing loss-of-function mouse models of autophagy achieved by cardiac-specific ATG5 or ATG7 ablation (Nakai et al., 2007; Taneike et al., 2010). In most cases, a functional macroautophagy machinery is needed for selective removal of damaged mitochondria, while ATG5 and ATG7 are essential genes for optimal autophagic responses (Youle and Narendra, 2011; Palikaras et al., 2018). Mice with temporally controlled cardiomyocyte-specific deletion of ATG5 exhibit left ventricular dilatation, with an accumulation of damaged mitochondria in the heart (Nakai et al., 2007; Taneike et al., 2010). ATG7-dependent activation of cardiac mitophagy recently has been reported to protect the myocardium from the metabolic stress of a high-fat diet (Tong et al., 2019). Therefore, interventions designed to stimulate autophagy in the cardiovascular system may prevent the accumulation of damaged mitochondria under cardiac stress and exhibit potential cardioprotective effects. Eisenberg et al. reported that the polyamine spermidine, delivered as a dietary supplement, can enhance cardiac autophagy/mitophagy, ameliorate age-related cardiac hypertrophy and preserve diastolic function in older mice (Eisenberg et al., 2016). Interestingly, a functional autophagy system in cardiomyocytes is required for the cardioprotective effects of spermidine (Eisenberg et al., 2016). Furthermore, genetic studies have demonstrated the key role of the Ser/Thr-kinase ULK1 in mediating mitophagy and early autophagosome formation (Wu et al., 2014). ULK1-regulated mitophagy is activated in response to energetic stress and may play an essential role in preserving mitochondrial function following myocardial ischemia (Saito et al., 2019).

The impact of PINK1 and Parkin has been extended in the cardiovascular system. In Drosophila models, genetic deletion of either PINK1 or Parkin can lead to mitochondrial dysfunction and poor cardiac contractibility (Guo, 2012; Bhandari et al., 2014). Genetic deletion of PINK1 in mice results in cardiac hypertrophy and progressive cardiac dysfunction (Billia et al., 2011). These mice also exhibit increased infarct size in response to ischemia-reperfusion injury (Siddall et al., 2013). Furthermore, PINK1-mediated mitochondrial quality control could be important during acute cardiac mitochondria stress following exhaustive exercise (Sliter et al., 2018). Mice with Parkin deletion demonstrate normal baseline cardiac phenotypes (Kubli et al., 2013; Piquereau et al., 2013), but exhibit an increased sensitivity to stress conditions from myocardial infarction (MI) or cardiac aging, along with a decline in cardiac mitophagy and accumulation of dysfunctional mitochondria (Hoshino et al., 2013; Kubli et al., 2013). It is worth mentioning those cardiac abnormalities, such as cardiomyopathy, arrhythmia, and sudden cardiac death, are still under investigation, though rare may occur in PD patients (Scorza et al., 2018). Therefore, under specific cardiac pathophysiological circumstances, PINK1 and Parkin may serve as a regulatory mechanism of mitophagy in the heart. Additional mitochondrial quality control pathways regulated by NIX and BNIP3 (Dorn, 2010), FUNDC1 (Zhang et al., 2017; Lampert et al., 2019) or general autophagy may compensate for the loss of PINK1/Parkin-mediated mitophagy. The functional significance of PINK1 and Parkin may require further investigation.

Recent progress also has linked mitophagy to the processes of mitochondrial dynamism, fission and fusion (Youle and Narendra, 2011; Palikaras et al., 2018; Pickles et al., 2018). Cell biological observations suggest that a group of fission and fusion proteins regulate mitochondrial morphology depending on the metabolic status (Mishra and Chan, 2016). Mitofusins, MFN1, and MFN2, as well as optic atrophy protein 1 (OPA1) promote the mitochondrial fusion (Mishra and Chan, 2016), whereas the dynamin-related protein 1 (DRP1) mediates mitochondrial fission (Friedman and Nunnari, 2014; Mishra and Chan, 2014, 2016) (Figure 1). MFN2 can be phosphorylated by the PINK1 kinase on the OMM, which facilitates Parkin translocation to promote mitophagy (Chen and Dorn, 2013). MFN2 deletion in mouse hearts disrupts mitophagic flux independent of its activity in fission/fusion regulation (Song et al., 2014). Mitochondrial fission, on the other hand, allows for mitochondrial fragmentation and has been suggested to regulate mitophagy (Rambold et al., 2011; Mishra and Chan, 2014, 2016). Several studies have demonstrated that genetic manipulation of DRP1 in mouse hearts can alter myocardial mitochondrial function and mitophagy (Kageyama et al., 2014; Song et al., 2015; Shirakabe et al., 2016b). Cardiomyocyte-specific homozygous deletion of DRP1 suppresses mitophagy, and leads to dilated cardiomyopathy in mouse models, while the heterozygous DRP1 knockout mice are more susceptible to ischemia/reperfusion injury (Ikeda et al., 2015). Furthermore, in the murine heart, DRP1 may mediate mitophagy in response to mitochondrial dysfunction under pressure overload, while haploinsufficiency of DRP1 exacerbates the progression of heart failure (Shirakabe et al., 2016b). The particular role for fission/fusion mediated mitophagy in healthy and in diseased hearts, as well as how PINK1 and Parkin participate in the regulatory mechanisms associated with mitochondrial fission/fusion is still under investigation (Kageyama et al., 2014; Song et al., 2015).

Cardiovascular disease imposes a huge burden worldwide, in terms of mortality, morbidity, and healthcare costs (Dokainish et al., 2017; Benjamin et al., 2019). Despite significant progress in understanding the pathophysiology of the disease, the prevalence of cardiovascular disease and its mortality rates remain high (Dokainish et al., 2017; Benjamin et al., 2019). Although preclinical studies suggest the potential benefit of mitochondria-targeted therapies in cardiovascular disease, it remains to be established whether the preservation of mitochondrial function through modulating mitophagy will result in improved clinical outcomes in patients (Murphy et al., 2016; Bravo-San Pedro et al., 2017). A better understanding of the regulatory mechanisms of mitophagy in the heart and its pathophysiologic role in cardiovascular disease are needed to develop effective mitophagy-targeted therapeutic agents and translate this innovative treatment strategy.

## Perspectives

Mitophagy serves as a critical mechanism to eliminate damaged mitochondria and is regulated by multiple mechanistically distinct pathways (Youle and Narendra, 2011; Palikaras et al., 2018). Cellular level studies have provided valuable insight into the signaling pathways regulating mitophagy, as well as mapping out how and when mitophagy occurs in a wide range of physiological and pathological conditions to counter cellular stressors such as ROS or damaged mitochondria (Youle and Narendra, 2011; Bravo-San Pedro et al., 2017; Palikaras et al., 2018). A better understanding of mitochondrial turnover mechanisms, with an improved focus on how these pathways might contribute to disease pathogenesis, should allow for the development of more efficient strategies to battle numerous pathological conditions associated with mitochondrial dysfunction.

Mitophagy is an important element of overall mitochondrial quality control. Defective mitophagy is thought to contribute to normal aging as well as various neurodegenerative and cardiovascular diseases (Youle and Narendra, 2011; Sun et al., 2016). In fact, aging by itself is a major risk factor for the pathophysiology of cardiovascular and neurodegenerative diseases (Eisenberg et al., 2016; Fivenson et al., 2017; Bonora et al., 2018). Increasing evidence suggests that mitophagy failure accelerates aging (Eisenberg et al., 2016; Sun et al., 2016; Fivenson et al., 2017; Bonora et al., 2018). Interestingly, a marked age-dependent decline in mitophagy has been observed in the hippocampus of the mouse brain (Sun et al., 2015), an area where new memory and learning are encoded. Similar effects have been noted in mouse models of HD (Sun et al., 2015). This strengthens the hypothesis that mitophagy might regulate neuronal homeostasis and that a decline in mitophagy might predispose to age-dependent neurodegeneration. Age-related mitochondrial function deterioration is underlined as a key feature of other diseases, such as obesity, diabetes, and cancer (Eisenberg et al., 2016; Sun et al., 2016; Fivenson et al., 2017; Bonora et al., 2018). Therefore, maintaining a healthy mitochondrial network via functional mitophagy may serve as an attractive therapeutic strategy in the treatment of a wide range of age-related diseases, and potentially regulate longevity.

The emergence of nutritional and pharmacological interventions to modulate autophagy/mitophagy and to serve as a potential therapeutic model is quite encouraging. Accumulation of ubiquitinated OMM proteins has been proposed to act as a signal for selective mitophagy (Youle and Narendra, 2011; Palikaras et al., 2018). As described above, ubiquitination of mitochondrial proteins is positively regulated, in part, by the E3 ubiquitin ligase, Parkin (Narendra et al., 2008, 2010; Matsuda et al., 2010; Youle and Narendra, 2011). In contrast, removal of ubiquitin is achieved by the action of resident mitochondrial deubiquitinases, most notably USP30, thereby acting to antagonize mitophagy (Bingol et al., 2014; Cunningham et al., 2015; Bingol and Sheng, 2016; Gersch et al., 2017). Inhibition of USP30 enzyme activity may provide an unambiguous avenue to pursue the role of mitophagy as a therapeutic target. In addition, the natural polyamine spermidine can preserve cardiac diastolic function in older mice by inducing mitophagy (Eisenberg et al., 2016). Inhibitors of mTOR can induce autophagy, and protect against various cardiac pathologies, and prolong lifespan in diverse species (Sciarretta et al., 2012; Yan et al., 2013; Dai et al., 2014; Ranek et al., 2019). Recently, three promising candidates that may stimulate and reinvigorate mitophagy process have been demonstrated to reduce the accumulation of amyloid-beta and phosphorylated tau in Alzheimer’s mouse brains (Fang et al., 2019). These compounds, including nicotinamide mononucleotide, urolithin A, and actinonin, can improve symptoms of AD and dementia symptoms in preclinical models (Fang et al., 2019). In addition, Tat-Beclin 1 peptide, derived from a region of the autophagy protein, beclin 1, can promote autophagy/mitophagy and improve mitochondrial function in heart failure animal models (Shirakabe et al., 2016b). Therefore, identifying more efficient and specific agents that can modulate the clearance of defective mitochondria are likely to have significant therapeutic benefits.

Recent advances have greatly expanded our knowledge of how mitophagy functions in health and disease. The magnitude and kinetics of mitophagy in various disease conditions, however, remains to be elucidated. Although mitophagy has many potential benefits, under certain conditions, uncontrolled or overactive mitophagy may disrupt organelles’ integrity and may prove detrimental to cell health (Liu et al., 2013; Saito and Sadoshima, 2015). Therefore, it is essential to elucidate how mitophagy collaborates with other cellular processes, such as autophagy, mitochondrial fission/fusion, and mitochondrial biogenesis to restore unhealthy mitochondria and maintain overall mitochondrial homeostasis. Furthermore, it is critical to determine when and to what extent manipulating mitophagy activity may regulate and keep mitochondria in health to prevent disease progression.

## Author Contributions

HL, RZ, and NS designed the outline of the review and wrote the draft of the review. JK, AM, and SO provided scientific comments and wrote part of the review.

## Conflict of Interest

The authors declare that the research was conducted in the absence of any commercial or financial relationships that could be construed as a potential conflict of interest.
